# Analysis of body posture of children with idiopathic scoliosis in the image Diers after the application of kinesiology taping application

**DOI:** 10.1186/1748-7161-9-S1-P15

**Published:** 2014-12-04

**Authors:** Zbigniew Śliwiński, Wojciech Kufel, Bartłomiej Halat, Beata Michalak, Danuta Śliwińska, Grzegorz Śliwiński

**Affiliations:** 1 Physiotherapy Center Zgorzelec, Zgorzelec, Poland; 2 Dresden University of Technology, Institute of Biomedical Engineering, Dresden, Germany

## Introduction

In the process of treating scoliosis essence of treatment is to maintain normal patterns of attitudes through appropriate antigravity muscle tone. Stimuli proper posture pattern in the period in which the child does not perform exercises provide appropriately selected and made corset. The use of dynamic slicing applications - Kinesiology Taping at the surface of the skin also stimulates the child to maintain proper muscle tone shaping correct posture.

## Material and method

Evaluated in a group of 40 children diagnosed with idiopathic scoliosis in age from 10 to 15 years residing in the treatment by the Fed at the Centre for Rehabilitation in Zgorzelec. Each child before treatment, the day of admission to the ward had made an assessment method Diers. Then an application of Kinesiology Taping. Used applications ligamentous took the form of V and were used on curves thoracic and lumbar scoliosis. Next, a re-image method Diers assessing mathematical representation of the body surface after the application of Kinesiology Taping.

## Results

The results obtained after the application of Kinesiology Taping show that the image of body posture changes, which record the images method Diers.

## Conclusions

Kinesiology Taping techniques are useful in the treatment of idiopathic scoliosis. Alter the tension of the skin and muscles make it easy to maintain the correct posture pattern

